# Analysis of DNA Methylation in a Three-Generation Family Reveals Widespread Genetic Influence on Epigenetic Regulation

**DOI:** 10.1371/journal.pgen.1002228

**Published:** 2011-08-11

**Authors:** Jason Gertz, Katherine E. Varley, Timothy E. Reddy, Kevin M. Bowling, Florencia Pauli, Stephanie L. Parker, Katerina S. Kucera, Huntington F. Willard, Richard M. Myers

**Affiliations:** 1 HudsonAlpha Institute for Biotechnology, Huntsville, Alabama, United States of America; 2 Duke Institute for Genome Sciences and Policy, Duke University, Durham, North Carolina, United States of America; Medical Research Council Human Genetics Unit, United Kingdom

## Abstract

The methylation of cytosines in CpG dinucleotides is essential for cellular differentiation and the progression of many cancers, and it plays an important role in gametic imprinting. To assess variation and inheritance of genome-wide patterns of DNA methylation simultaneously in humans, we applied reduced representation bisulfite sequencing (RRBS) to somatic DNA from six members of a three-generation family. We observed that 8.1% of heterozygous SNPs are associated with differential methylation in *cis*, which provides a robust signature for Mendelian transmission and relatedness. The vast majority of differential methylation between homologous chromosomes (>92%) occurs on a particular haplotype as opposed to being associated with the gender of the parent of origin, indicating that genotype affects DNA methylation of far more loci than does gametic imprinting. We found that 75% of genotype-dependent differential methylation events in the family are also seen in unrelated individuals and that overall genotype can explain 80% of the variation in DNA methylation. These events are under-represented in CpG islands, enriched in intergenic regions, and located in regions of low evolutionary conservation. Even though they are generally not in functionally constrained regions, 22% (twice as many as expected by chance) of genes harboring genotype-dependent DNA methylation exhibited allele-specific gene expression as measured by RNA-seq of a lymphoblastoid cell line, indicating that some of these events are associated with gene expression differences. Overall, our results demonstrate that the influence of genotype on patterns of DNA methylation is widespread in the genome and greatly exceeds the influence of imprinting on genome-wide methylation patterns.

## Author Summary

DNA methylation is a dynamic epigenetic mark that is essential for mammalian organismal development. DNA methylation levels can be influenced by environment, a chromosome's parental origin, and genome sequence. In this study, we evaluated the impact that DNA sequence has on DNA methylation by analyzing methylation levels in a three-generation family as well as unrelated individuals. By following DNA methylation patterns through the family along with nearby SNPs, we found that allelic differences between chromosomes play a much larger role in determining DNA methylation than the parental origin of the chromosome, indicating that DNA sequence has a larger impact on DNA methylation than gametic imprinting. We also found that allelic differences in DNA methylation found in the family can also be observed in unrelated individuals. In fact, the majority of variation in DNA methylation can be explained by genotype. Our results emphasize the importance of genome sequence in setting patterns of DNA methylation and indicate that genotype will need to be taken into account when assessing DNA methylation in the context of disease.

## Introduction

Methylation of the 5 carbon of a large number of cytosines in the genome is necessary in mammalian development [1]. Aberrant patterns of DNA methylation have been reported in a wide variety of human diseases, including cancer [2], [3], psychiatric disorders [4], autoimmune diseases [5] and diabetes [6]. Some of these patterns are indicative of underlying functional changes that have occurred during disease progression and shed light on genes involved in pathogenesis. One of the most fascinating discoveries surrounding DNA methylation is the observation that two homologous chromosomes can be differentially methylated.

Differential methylation of homologous chromosomes can be the result of epigenetic phenomena such as gametic imprinting [7], [8] or X chromosome inactivation [9], [10]. DNA sequence, or genotype, may also play a role in establishing differential methylation, as a few well-established cases have been identified in which a locus' DNA methylation state clearly depends on an individual's DNA sequence [11], [12]. Recent advances in DNA sequencing technology have opened the door to exploring differential methylation on homologous chromosomes with high accuracy and detail. It is now possible to examine the prevalence of genetic versus epigenetic causes of differential methylation with unprecedented precision and thoroughness.

To distinguish the impact of gametic imprinting vs. genotype on DNA methylation, the inheritance patterns of alleles along with corresponding methylation levels should be observed. Recent studies have suggested that the majority of differential DNA methylation on homologous chromosomes is sequence-dependent and not the result of gametic imprinting, as the same allele has the same influence on DNA methylation in unrelated individuals [13]–[15]. However, to differentiate definitively between genetic inheritance and imprinting, analysis of DNA methylation in primary tissues from a family is necessary. Analysis of a family allows for the determination of a SNP's parental origin along with inheritance patterns of DNA methylation levels and therefore permits the direct examination of genetic and epigenetic mechanisms of differential methylation. By analyzing DNA methylation in a family, the impact of alleles verses the impact of a chromosome's parental origin on the inheritance of methylation can be clearly resolved. Surveying DNA methylation in multiple related and unrelated individuals also enables quantitative estimation of the effect of genotypic variation on DNA methylation levels.

Examination of DNA methylation patterns in twins has indicated that related individuals exhibit more similar DNA methylation than unrelated individuals, implying that genetics plays a role in establishing DNA methylation patterns. Baranzini et al. [16] observed a striking similarity in DNA methylation patterns between monozygotic twins that was not seen between unrelated individuals. In addition, Kaminsky et al. [17] found that monozygotic twins share similar DNA methylation patterns compared to dizygotic twins; however, they suggest that this similarity may be due to epigenetic events in the zygote as opposed to genetic relatedness. Considering the promise of DNA methylation as a disease biomarker [18], it is important to determine the influence that genome sequence has on this epigenetic mark.

To determine the genetic contribution to DNA methylation, we quantified DNA methylation in non-immortalized peripheral blood leukocytes from a three-generation family. We used reduced representation bisulfite sequencing (RRBS) [19] to achieve single molecule resolution of DNA methylation for a large number of CpG dinucleotides distributed throughout the human genome. We were able to find SNPs by sequencing and directly observing DNA methylation on the same homologous chromosome. Our results show that differential methylation of homologous chromosomes is prevalent, transmitted through families in a genotype-dependent manner, and linked with allele-specific gene expression.

## Results

### Allele-specific DNA methylation discovery by RRBS is reproducible

To discover differential methylation on homologous chromosomes, we used reduced representation bisulfite sequencing (RRBS) [19], which can be used to measure the DNA methylation state in a subset of the genome in many samples. Because RRBS uses bisulfite treatment, it detects both 5-methylcytosine and 5-hydroxymethylcytosine [20]. Thus, the allelic differences identified by the method can be differences in 5-methylcytosine or 5-hydroxymethylcytosine. However, alleles that differ in the ratio between levels of 5-methylcytosine and 5-hydroxymethylcytosine are not detectable. We refer to the combination of these marks as DNA methylation.

**Figure 1:**
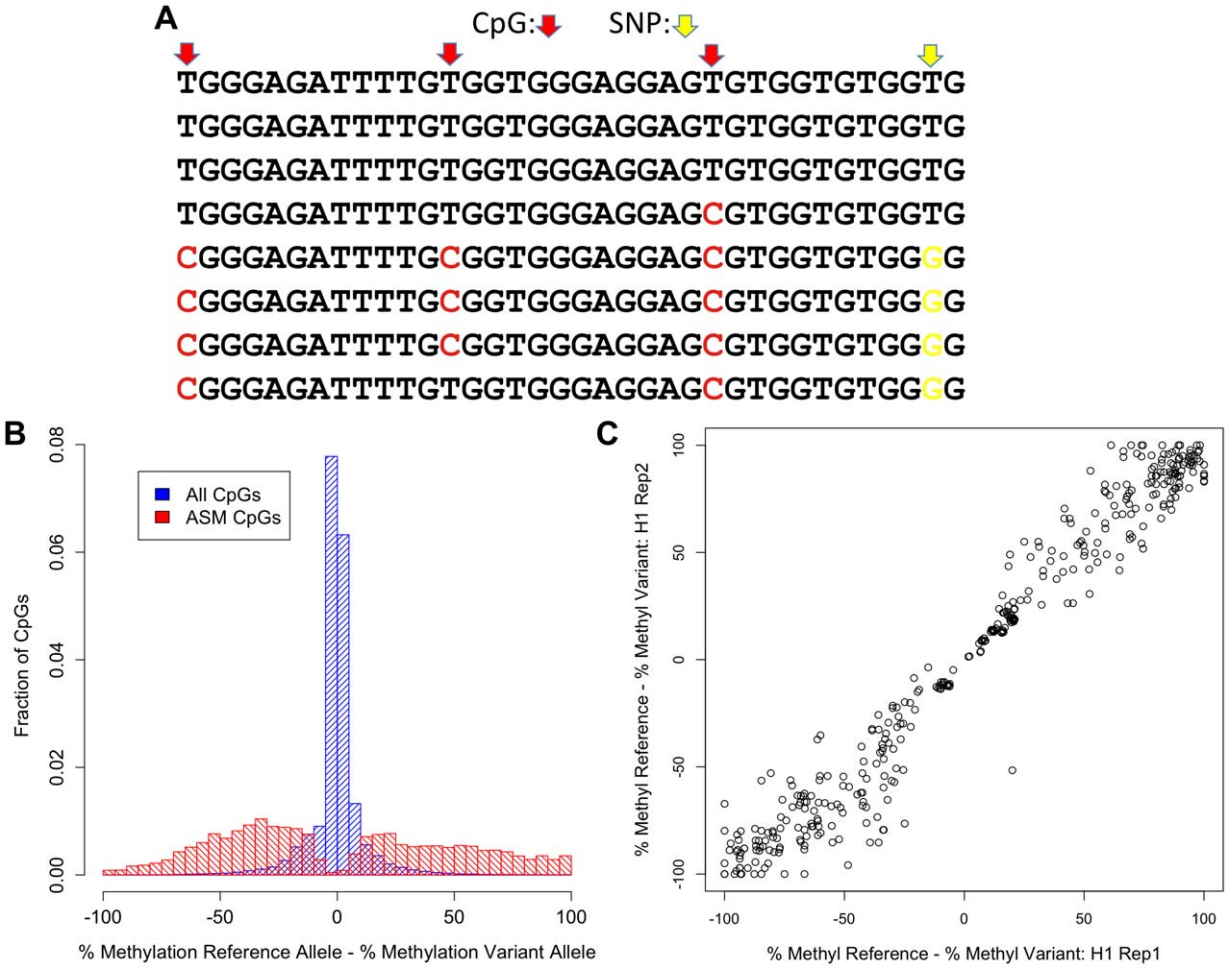
Discovery of ASM by reduced representation bisulfite sequencing. (A) Example of bisulfite sequencing reads from a region with a SNP (T/G; yellow) that is associated with methylation levels. Methylated cytosines, which appear as C's in the bisulfite reads, are shown in red. (B) Distribution of the difference in percent methylation of the reference allele and the variant allele is shown for all CpGs that are nearby a SNP (blue) and ASM CpGs that do not involve CpG-disrupting SNPs (red). (C) The difference in percent methylation of the reference allele and the variant allele for ASM events not involving a CpG-disrupting SNP from two biological replicates of the hESC H1 line.

We used the Illumina Genome Analyzer IIx (GAIIx) to sequence 36 base pair ends of bisulfite-treated MspI digestion fragments ranging in length from 40 to 120 bp. For each sample, we assayed approximately 1 million CpG dinucleotides with at least 10 sequencing reads, and calculated the percent of reads that are methylated at each CpG. This type of deep sequencing also identifies DNA sequence variants in the fragments for which we are measuring methylation (see Materials and Methods). Detecting these SNPs in the bisulfite sequence reads allows direct determination of whether a particular allele is found in *cis* with methylated or unmethylated CpGs (Figure 1A). Bisulfite treatment obscures C to T SNPs in forward strand reads and G to A SNPs in reverse strand reads, because a C to T mismatch in forward strand reads could represent an unmethylated cytosine and cannot be unambiguously called a SNP. We do not analyze possible C to T SNPs in our sequence reads because they cannot be identified. These SNPs account for approximately 30% of human SNPs; thus, most SNPs remain observable.

We first measured DNA methylation on different alleles in the human embryonic stem cell line H1 [21]. The majority of alleles show no *cis* association with DNA methylation. At a 5% false discovery rate (FDR), 5.4% (1,340 of 24,979) of autosomal heterozygous SNPs are associated with allele-specific methylation (ASM). In total, 1,340 SNPs are associated with ASM at 1,574 CpGs. Because a SNP can be linked to multiple CpGs and multiple SNPs can be linked to the same CpG, we were able to identify 1,937 ASM events in this cell line (all ASM data can be found in Dataset S1; Table S1 provides a summary of ASM events in each sample, including the number of heterozygous SNPs called). Of the 1,937 ASM events, 573 (29.6%) involve a SNP that mutates a CpG. These cases represent a trivial mechanism for generating ASM because there is no longer a CpG to methylate when the CpG-disrupting allele is present; however, they are functional variants in that they result in a change in the methylation status of a locus.

The average difference in percent methylation between alleles is 59.8% for ASM events that do not involve CpG disrupting SNPs (Figure 1B). Less than 8% of ASM events exhibit greater than a 95% difference in percent methylation, which suggests that most changes in genotype induce more subtle changes in the frequency of DNA methylation than complete reversal of CpG methylation. These quantitative differences in percent methylation between alleles are highly reproducible. In two separate growths of the H1 cell line, the correlation of the difference in percent methylation between the reference and variant allele for all ASM events in both replicates is 0.98 (Figure 1C). These results show that measurements of the quantitative differences in percent methylation associated with SNPs are highly reproducible.

### ASM associates with X chromosome inactivation

One of the two X chromosomes in women are randomly silenced in each cell during development, and DNA methylation at many sites has been shown to exist specifically on alleles from the inactive X [10]. To assess our ability to observe inactivation of the X chromosome and thus validate the ASM approach taken here, we analyzed ASM in four clonal cell lines derived from single cells of the EBV-transformed lymphoblastoid line GM12878. Gene expression patterns indicate that two of these cell lines silence the paternal X chromosome, while the other two cell lines silence the maternal X chromosome (Table S2; K.S.K. and H.F.W., manuscript in preparation). We observed reproducible patterns of ASM on the X chromosome (Figure S1). Clones with an inactivate X chromosome from the same parental lineage exhibited the same allelic bias for every ASM event on the X chromosome (e.g., either the variant allele was more methylated in each clonal line or the reference allele was more methylated in each clonal line). When comparing any maternal X-inactive clone with any paternal X-inactive clone, an average of 59.7% of ASM events on the X chromosome switch the allele that is most often methylated, which is consistent with the pattern of inactivation. Of the genes nearby the ASM events that are consistent with inactivation, six were previously assayed for allelic expression [22] and all six were identified as being inactivated. Because inactivation is not complete across the chromosome, we do not expect all ASM to switch the most often methylated allele when the inactive X chromosome is switched. These results demonstrate that our method is sensitive to X inactivation in single cell clones; however, many ASM events on the X chromosome are driven by allele status rather than by parental origin.

### ASM is prevalent in a three-generation family

**Figure 2:**
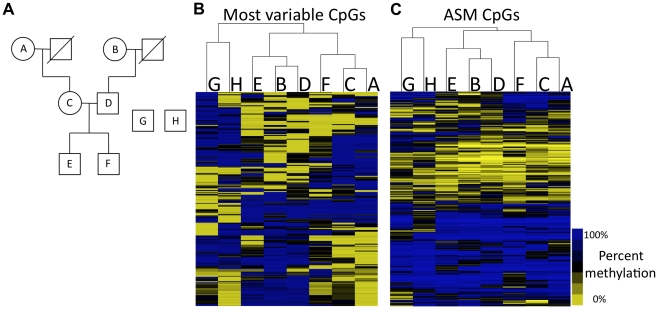
Methylation patterns recapitulate family relationships. (A) Pedigree is shown for family members analyzed (A–F) as well as unrelated individuals G and H. (B) Hierarchical clustering of the most variable CpGs across all of the individuals. (C) Hierarchical clustering of CpGs that exhibit ASM in at least one individual. Letters at the top of each cluster refer to letters in the pedigree.

To determine the prevalence of ASM throughout the human genome and to deconvolute whether it is dependent on DNA sequence variants or parental origin imprinting, we applied RRBS to DNA extracted from leukocytes in fresh blood from six members of a three-generation family as well as two unrelated individuals (Figure 2A). We collected data for more than 950,000 CpGs that had at least ten sequencing reads in each family member. Overall, 859,531 CpGs had at least 10 sequencing reads in all samples and 668,545 had at least 20 sequencing reads in all samples. Leukocytes from fresh blood are comprised of a heterogeneous population of cells, consisting primarily of neutrophils, lymphocytes and monocytes. It has been shown that the relative proportion of each cell type does not considerably affect DNA methylation levels [23]. Consistent with this observation, we found that the patterns of DNA methylation are strikingly similar between the family members. The average correlation in percent methylation of all autosomal CpGs assayed between any two family members is 0.985. All of the samples assayed were highly similar, indicating that there were no systematic biases in DNA isolation, library preparation or DNA sequencing.

Analysis of the CpGs that vary within the eight individuals reveals that DNA methylation patterns recapitulate relatedness. We clustered individuals based on the methylation status of the top 237 most varying autosomal CpGs across all samples. The resulting tree, based on hierarchical clustering, is shown in Figure 2B. The two unrelated individuals are separated from the family members and serve as an out-group. Genetically unrelated pairs of family members (e.g., maternal grandmother and father, and father and mother in the middle generation) are also separated in the tree. The closest relationships in DNA methylation patterns exist between the grandmothers and their children and the methylation patterns in the grandchildren are mixtures of their parents' methylation patterns. These results indicate that DNA methylation levels can capture the relatedness of individuals, suggesting that there is a strong genetic component to DNA methylation levels.

We identified an average of 1,702 ASM events for each member of the family. When individuals are clustered on CpGs that exhibit ASM in at least one individual (Figure 2C), the same relationships are observed as those shown in Figure 2B. To look at features of CpGs exhibiting ASM, we focused on 2,391 autosomal ASM events that we identified in at least two of the six family members. 42.6% (1,018) of these ASM events are SNPs that mutate a CpG. The high prevalence of CpG-disrupting SNPs is not due to higher sequence coverage (Table S3). Our analysis showed that SNPs that disrupt CpGs tend to lie in intergenic regions that are not CpG islands. Only 21.9% of CpGs that are disrupted by SNPs are found in CpG islands, while 65% of all CpGs queried by RRBS reside in CpG islands (*P*<2.2×10^−16^; Fisher's exact test). CpGs that are disrupted by SNPs are over-represented in intergenic regions, as 44.1% are at least 2 kilobase pairs (kb) away from the nearest gene, compared to 22% of all CpGs assayed by RRBS that are at least 2 kb away from the nearest gene (*P*<2.2×10^−16^; Fisher's exact test). SNPs that mutate a CpG are often linked to the methylation status of other CpGs in the immediate vicinity. More than 45% of CpG-mutating ASM SNPs exhibit ASM associated with at least one other CpG within 36 base pairs, which represents a 5.5-fold increase in the chance that a CpG with a CpG-disrupting SNP in the same read shows allele specificity (P<2.2×10–16; Fisher's exact test). These data demonstrate that, while CpG-disrupting SNPs alter DNA methylation at their particular position due to a DNA sequence difference, they also influence the methylation of nearby CpGs. This is further evidence that there is a strong genetic effect on the regulation of DNA methylation.

**Figure 3:**
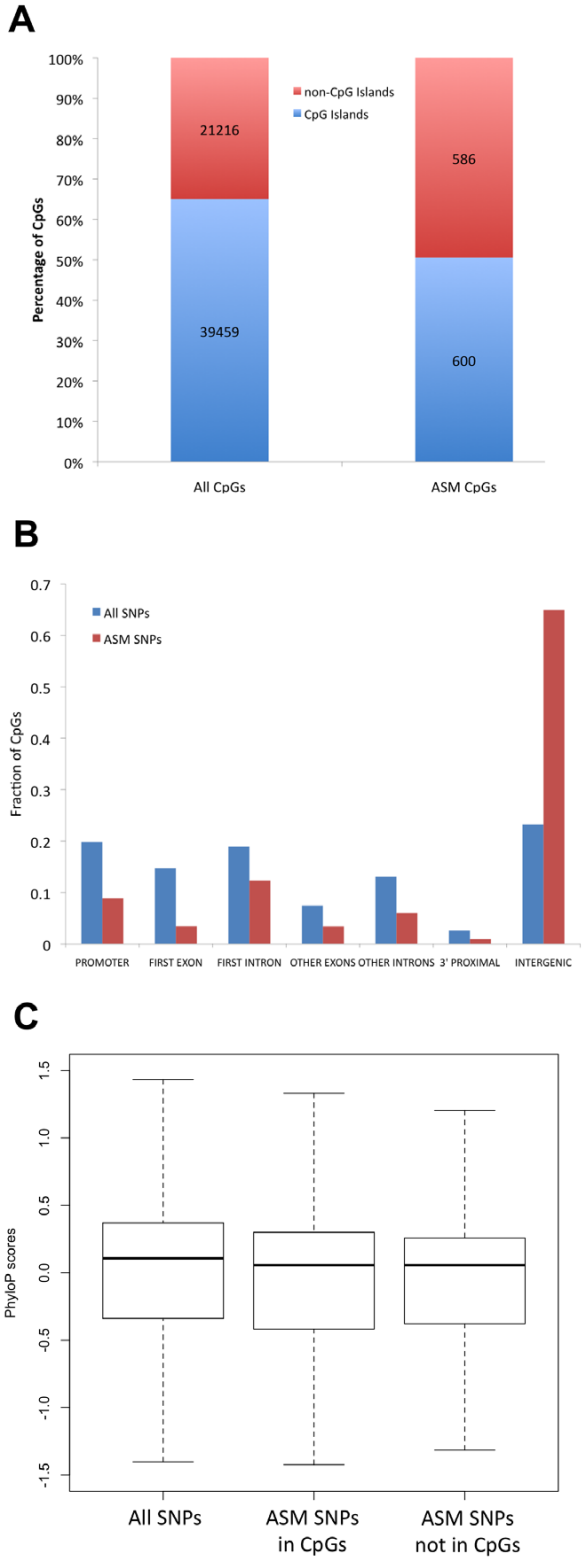
ASM is often found in regions that are outside of CpG islands, away from genes, and that exhibit low evolutionary conservation. (A) The percentage of CpGs found in CpG islands is shown for all CpGs and ASM CpGs. (B) The distribution of genomic locations with respect to genes is shown for all CpGs (blue) and ASM CpGs (red). (C) The distribution of evolutionary conservation scores (phyloP) for intergenic SNPs is shown as a boxplot for all SNPs near CpGs, ASM SNPs in which a SNP mutates a CpG, and ASM SNPs in which a SNP does not mutate a CpG. The midline represents the median conservation score and the box represents the 25% and 75% quantiles.

ASM events that do not involve a mutated CpG are under-represented in CpG islands and are most often located in intergenic regions, as were CpGs disrupted by SNPs. Only 49.4% of CpGs that exhibit ASM are present in CpG islands, even though 65% of CpGs with nearby SNPs assayed by RRBS are in islands (Figure 3A; *P*<2.2×10^−16^: Fisher's exact test). Figure 3B shows the distribution of gene features for CpGs that exhibit ASM. The majority of ASM CpGs are present in intergenic regions, even though most CpGs with nearby SNPs assayed by RRBS are present in promoters, first introns or first exons. There is a 3.5-fold increase in the fraction of CpGs that reside in intergenic regions (*P*<2.2×10^−16^; Fisher's exact test). The overall trends of where ASM events are located in the genome are not the result of differences in coverage between these categories of SNPs (Table S3). The tendency for ASM CpGs to be located outside of CpG islands and further away from genes may indicate that ASM events occur in regions under less selective pressure.

Because ASM tends to occur in regions without clear regulatory function, we looked at evolutionary constraint at these sites by analyzing mammalian alignments. ASM events involving SNPs that disrupt CpGs and SNPs that do not disrupt CpGs were found more often outside of CpG islands and in intergenic regions. We therefore assayed both sets and determined the level of evolutionary conservation in each group. We found significantly less conservation in CpG-disrupting ASM events (*P* = 0.008; Wilcoxon test) and non-CpG-disrupting ASM events (*P* = 0.002; Wilcoxon test) compared to all assayed regions that contained SNPs. To determine if this reduction in conservation could be explained by the overabundance of ASM events in intergenic regions, we repeated the conservation analysis using only SNPs in intergenic regions. We found that even within intergenic regions, SNPs associated with ASM were significantly less conserved than all intergenic SNPs assayed (Figure 3C). This was true for both CpG-disrupting ASM events (*P* = 0.009; Wilcoxon test) and non-CpG-disrupting ASM events (*P* = 0.007; Wilcoxon test). These data are consistent with the hypothesis that there is evolutionary constraint on DNA methylation levels, as genetic variants that affect DNA methylation tend to lie in regions under less selective pressure.

### Genotype influences allelic methylation more often than parental origin

**Figure 4:**
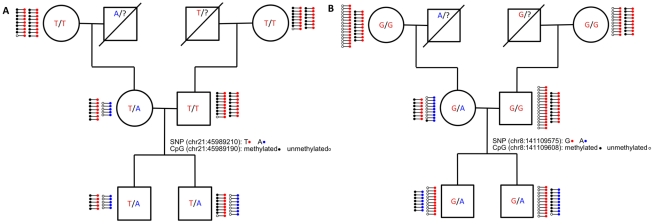
Inheritance of ASM in the family. The pedigree of the family is shown with their allele status at (A) chromosome 21 position 45,989,210, which is in an intron of c21orf29, and (B) chromosome 8 position 141,109,575, which is in an intron of TRAPPC9. Next to the assayed individuals, each line with two dots on the ends represents 5 sequence reads. The dot on the left side of each line represents the methylation status of the nearby CpG (black: methylated; white: unmethylated). The dot on the right side of the each line represents the allele status of the SNP (blue: reference allele and red: variant allele).

By carrying out RRBS in a family, we were able to follow SNPs through each generation and determine whether ASM is associated with the identity of the SNP or with parental origin. To distinguish these modes of transmission, we first had to identify SNPs with unambiguous inheritance in the pedigree that switched parental origin (e.g., those SNPs that have a maternal origin in one generation and a paternal origin in the next). We then identified those SNPs that were associated with ASM in both parent and child. We identified 432 autosomal ASM events in which the variant allele switched parental origin in the family. We then eliminated the 341 of these ASM events that were due to SNPs that mutated a CpG, as these are necessarily associated with the identity of the SNP. Only 7 (7.7%) of the remaining 91 ASM events displayed a methylation pattern that followed parental origin. The methylation patterns of the other 84 ASM events depended on the DNA sequence and an example is shown in Figure 4A. These results indicate that, in most cases, an allele's sequence plays a larger role in determining DNA methylation than an allele's parental origin.

While the majority of ASM events depend on the underlying allelic sequence, we found seven autosomal ASM events that are consistent with a genetic imprinting mode of transmission. These events occur in five distinct loci, most of which are intergenic. The introns of OVOS1 and TRAPPC9 both contain parent-of-origin ASM events, while the other three loci are at least 20 kb from the nearest gene. Figure 4B shows ASM of TRAPPC9 (also know as NIBP), which is involved in neuronal NF-κB signaling [24] and is thought to have a maternal effect on height [25]. The TRAPPC9 SNP mutates a CpG, which exhibits ASM that follows the sequence of the allele as expected. However, the CpG located 32 bp away is unmethylated on the variant allele in the mother (which came from her father) and is methylated on the variant allele in both sons. The RRBS data suggests that parent-of-origin ASM may extend further in this region, as CpGs extending 65 bp upstream and 125 bp downstream of the SNP are all close to 50% methylated in every member of the family. The transcription start site of KCNK9, a known maternally imprinted gene [26], resides downstream of TRAPPC9 more than 350 kb away from the SNP. It is possible that methylation of a TRAPPC9 intron is involved in silencing KCNK9. The TRAPPC9 region represents a candidate for maternal imprinting, which provides supporting evidence for the locus' role in maternal effect on height.

Because genotype-dependent differences in DNA methylation were far more prevalent than parental origin-dependent differences, we built a general model of DNA methylation and genotype that was able to incorporate information from homozygous individuals. We built a linear model of the relationship between DNA methylation and genotype that assumes that a CpG's methylation level in a heterozygous individual is halfway between the level of methylation in an individual homozygous for one allele and an individual homozygous for the other allele. When the model is constructed for the top 2,900 most variable CpGs in the family that have detectable SNPs nearby, 602 (20.8%) showed significant genotype contributions and 370 (12.8%) were still significant after multiple hypothesis correction. Even with a small sample size of six, genetic association with DNA methylation is detectable for a large number of loci. These results, together with ASM data, suggest that DNA sequence plays a major role in determining inter-individual differences in DNA methylation levels.

### ASM is also observed in unrelated individuals

Some of the genetically linked ASM events found in the family may be dependent on genetic background and specific to the particular family studied, while other ASM events may be common to many individuals. To determine the persistence of ASM in different genetic backgrounds, we analyzed two unrelated individuals as well as the lymphoblastoid cell line GM12878. We could test 40 of the 84 sequence-dependent autosomal regions that we observed in the family because at least one unrelated individual was heterozygous at the same SNP as that observed in the family. 30 of those 40 regions (75%) were found to be allele-specific in the unrelated individuals as well. In every case, the more often methylated allele in the family was also more often methylated in the unrelated individuals. We also found that the CpGs in the TRAPPC9 locus discussed above were also methylated between 39% and 57% in the unrelated individuals, which is consistent with parental origin imprinting at this locus. These results indicate that many ASM events that we observed in the family are present in other genetic backgrounds.

For a broader view of the influence of genotype on DNA methylation, we used the linear model of DNA methylation and genotype discussed above to predicted DNA methylation levels in the two unrelated individuals. In both individuals, the model was able to predict methylation levels with high accuracy based on each individual's genotype (*R^2^* = 0.82 and 0.83). The ability of the linear model, which was trained on the family, to predict DNA methylation in unrelated individuals accurately shows that the relationship between DNA sequence and methylation levels is consistent among individuals.

While the influence of genotype could be observed across individuals in the same tissue, we sought to determine whether the impact of DNA sequence on DNA methylation could be observed in different tissues from the same individual. We analyzed DNA extracted from kidney and skeletal muscle of a third unrelated individual. There were 1,521 ASM events in the kidney sample and 1,332 ASM events observed in the skeletal muscle sample. Of the 984 ASM events observed in the kidney with sufficient coverage in both samples, 834 (84.8%) were also allele-specific in skeletal muscle. For all 834 overlapping ASM events, the more often methylated allele was identical in the kidney and skeletal muscle samples. If CpG disrupting SNPs are removed from the analysis, there were 931 ASM events in the kidney sample and 790 events observed in the skeletal muscle sample. Of the 614 ASM events observed in the kidney with sufficient coverage in both samples, 445 (72.5%) were also allele-specific in skeletal muscle. These results indicate that allelic differences in DNA methylation can be observed across different tissues in the same individual at a high frequency.

### ASM associates with gene expression differences

**Figure 5:**
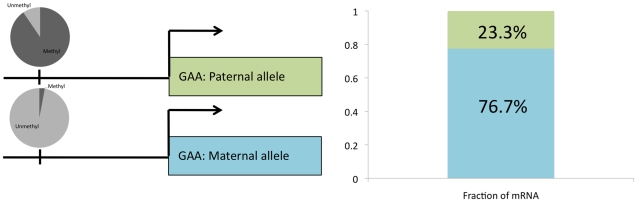
Allele-specific gene expression overlaps with ASM. Methylation of upstream CpG on each allele is shown in a pie chart with dark gray representing the fraction of CpG sites that is methylated and light gray representing the fraction unmethylated (left side). The fraction of mRNA that comes from each allele is shown for GAA (right side).

To determine whether ASM events are indicative of functional gene regulatory differences, we compared ASM with allele-specific gene expression (ASE; T.E.R. et al., manuscript in preparation) in the original lymphoblastoid line GM12878. Of the autosomal genes that have an ASM event within 5 kb of the transcription start site, we found that 21.7% (18 out of 83) of the autosomal genes with sufficient RNA-seq read depth (greater than 25 reads that cover SNPs) show ASE (Table S4). This represents more than a two-fold enrichment (*P* = 4.2×10^−4^; hypergeometric test) over an overall rate of 9.2% (594 out of 6464) of ASE for all autosomal genes with sufficient read depth. Data for the gene encoding acid alpha-glucosidase (GAA) are summarized in Figure 5. GAA, which is involved in the degradation of glycogen to glucose and is associated with Pompe's disease [27], showed 3% methylation on the maternally inherited copy of chromosome 17 and 89% methylation on the paternally inherited copy; RNA-seq showed that 76.7% of the transcripts came from the maternal copy of chromosome 17. TRAPPC9 and OVOS1 did not harbor SNPs with sufficient read depth in the RNA-seq data to determine allele-specificity. These results indicate that genes exhibiting ASM are enriched for gene expression differences between alleles.

### Independent validation of ASM and ASE

**Figure 6:**
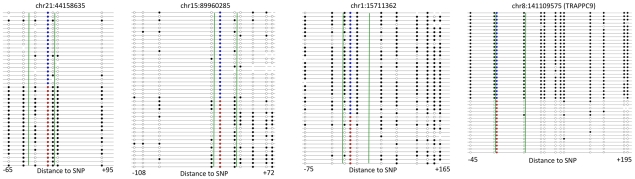
ASM extends past regions assayed by RRBS. Results from Sanger sequencing of bisulfite PCR clones are shown for four loci where the chromosomal location indicates the position of the SNP. Each line represents one read where black circles represent methylated CpGs, white circles represent unmethylated CpGs, blue circles represent the reference allele and red circles represent the variant allele. Green lines demarcate the region assayed by RRBS. The distance from the SNP to the boundaries of the region is shown below each loci.

We sought to validate the next generation sequencing results by performing Sanger sequencing on a small number of loci. To investigate ASM, we performed bisulfite PCR, cloning and Sanger sequencing of four loci from family member C (Figure 6). All four loci exhibited ASM in the Sanger sequencing data. We also observed that the allele-specificity of DNA methylation extended beyond the region assayed by RRBS. In two cases, (chr21:4415835 and chr8:141109575) allele-specificity was found across the entire region. In the other two examples, half of the assayed region exhibited allele-specificity, while the other half of the region was similar between the two alleles. These results indicate that ASM identified in the RRBS data is also observed with Sanger sequencing and that patterns of ASM are complex.

To look further at extended ASM, we analyzed SNPs within 200 bp of each other and found that adjacent SNPs were often concurrently associated with ASM. For example, in family member A 655 ASM SNPs have another SNP detected within 200 bp and 455 (71.4%) of these SNPs also exhibit ASM. On average across the family members, 76.3% of SNPs adjacent to ASM SNPs were also associated with ASM themselves. These results show that ASM can be seen across extended regions.

To validate the next generation ASE findings, we cloned and Sanger sequenced genomic DNA and cDNA from GM12878 for six loci. Using the ratio of alleles observed in the genomic DNA as a background, we found that five (GAA, KCNQ10T1, HLA-DPB2, LOC654433 and LOC253039) loci exhibited significant allele biased expression with the bias coming from the expected parental chromosome. ZNF132 showed a bias in the expected direction (69% from the allele predicted to be higher expressed); however, the number of reads (16), was too low to call the bias significant. Both ASE and ASM validation results show that the allele-specificity observed with our next generation sequencing approaches are replicated with an independent technique.

## Discussion

Using short-read, ultra high-throughput DNA sequencing, we identified reproducible quantitative differences in DNA methylation between alleles. When we observe allele-specific DNA methylation, the differences in methylation levels between homologous chromosomes are rarely completely reversed. The abundance of subtle differences indicates that some alleles influence the propensity of DNA methylation within a cell population, but do not completely exclude or cause methylation. ASM events were found outside of CpG islands and were highly enriched in intergenic regions. Because ASM tends to occur in regions without clear regulatory function, we looked at evolutionary constraint at these sites. ASM tends to be found in genomic locations with significantly low levels of evolutionary conservation. This result is consistent with the hypothesis that there is selective pressure to maintain DNA methylation, as we found that most alleles that affect DNA methylation exhibit less evolutionary constraint. Selection may be acting on the underlying events that lead to DNA methylation or directly on the ability to methylate a particular sequence, as is the case with SNPs that mutate CpGs.

We found that DNA methylation levels could distinguish family members from unrelated individuals and capture family relationships. By analyzing six members of a three-generation family, we were able to follow the patterns of inheritance of both DNA methylation and the SNPs associated with methylation levels directly. When the parental origin of a SNP switched between generations, the vast majority of ASM events were genotype-dependent and followed a particular sequence variant rather than parental origin. These results show that DNA sequence plays a larger role in establishing DNA methylation patterns than do parental origins. We found that the majority of the ASM events seen in the family could also be observed in other individuals, indicating that the ASM events observed are not specific to the family described here and that the alleles have the same influence on DNA methylation in different genetic backgrounds.

The strong association between genotype and DNA methylation indicates that genetics plays a prominent role in the establishment of DNA methylation patterns. Our data supports a non-Lamarckian model of evolution, where genetic variants, as opposed to environment, shape epigenetics [28]. These genetic variants may not lead directly to phenotypic differences, but may cause phenotypic variability through changes in epigenetic states.

While ASM events could be observed across individuals in the same cell type, we also observed a concordance of ASM between tissue types. The fact that we see a strong overlap between ASM in different tissues indicates that these allelic differences are most likely due either to shared gene regulatory events that occur early in development or an inherent property of DNA sequence that directly affects the propensity of DNA methylation. The prevalence of ASM in different tissues brings up the possibility that methylation at these loci are directly inherited with the haplotype through the germline. While the prevailing model of DNA methylation would suggest that methylation patterns are erased during gamete formation and just after fertilization [29], the possibility exists that DNA methylation is being constantly maintained for these loci in an allele-specific manner. Our data are consistent with both the re-establishment of allelic methylation during development and the direct transmission of DNA methylation in the germline, and cannot distinguish between these modes of transmission.

There may be alleles that influence the conversion of 5-methylcytosine to 5-hydroxymethylcytosine by the TET family of enzymes. Because the use of bisulfite sequencing does not distinguish between modifications, these alleles would not be detectable in our data. It will be interesting to determine if newly described genome scale methods [30]–[32] will be able to identify allelic differences in 5-methylcytosine and 5-hydroxymethylcytosine separately.

## Materials and Methods

### DNA extraction

We extracted high molecular weight genomic DNA from 8 ml of blood from each individual. The buffy coats from each sample were isolated by centrifugation. Buffy coat was gently mixed and incubated for 30 minutes with lysis buffer at room temperature (0.32 M Sucrose, 10 mM Tris-HCl pH 7.5, 5 mM MgCl_2_ and 1% Triton X-100), then centrifuged at 4°C at 2500 rpm for 20 minutes. The supernatant was discarded, and the pellet was vortexed with 20 ml lysis buffer, then centrifuged at 4°C at 2,500 rpm for 20 minutes. The supernatant was discarded and the pellet was incubated in 5 ml guanidine isothiocyanate buffer on a shaker for 25 minutes (5 M Guanidine thiocyanate, 25 mM sodium acetate, 0.84% beta-mercaptoethanol). 5 ml 4°C isopropanol was added and the sample was inverted gently until precipitate appeared. Samples were then incubated at −20°C for at least one hour, and centrifuged at 4°C at 2,500 rpm for 20 minutes. The supernatant was then discarded and 500 µl T10E.2 (10 mM Tris-HCl pH 7.4, 2 mM EDTA pH 8.0), 50 µl 3 M sodium acetate pH 5.2, and 1 ml 100% ethanol were added and mixed and the DNA was precipitated by centrifuging at 12,000 rpm for 30 seconds. The supernatant was discarded and the pellet was washed with 500 µl 70% ethanol and centrifuged at 12,000 rpm for 30 seconds. The supernatant was discarded and the DNA was allowed to air-dry for 10 minutes. The genomic DNA pellet was resuspended in 1 ml T10E.2 buffer by gentle vortexing.

For GM12878 and H1 hESC, cells were grown according to ENCODE standards [33]. To generate the clonal cell lines, GM12878 was cultured according to the ENCODE protocol. At the time of culturing, the original GM12878 cell line was heavily skewed (92%) towards the paternally inherited inactive X chromosome [34]. To obtain pure populations of cells with an inactivated maternal X chromosome (Xi^mat^) or an inactivated paternal X chromosome (Xi^pat^), single cell clones of GM12878 were obtained by serial dilution. Each selected clone was tested for complete nonrandom inactivation by a PCR-based SNaPshot expression assay [22] using heterozygous SNPs in monoallelically expressed X-linked genes (Table S2; K.S.K. and H.F.W., manuscript in preparation). Four clones (two with Xi^mat^ and two with Xi^pat^) were chosen for further study. For all cell lines, DNA was extracted from cell pellets using a DNeasy kit (Qiagen) according to the manufacturer's instructions. Kidney and skeletal muscle genomic DNA from the same donor was purchased from BioChain (Hayward, CA). Genomic DNA was quantified using fluorescent DNA binding dye and a fluorometer (Invitrogen Quant-iT dsDNA High Sensitivity Kit and Qubit Fluorometer).

### Reduced representation bisulfite sequencing

Reduced representation bisulfite sequencing (RRBS) was performed as described [35]. Briefly, 1 µg genomic DNA was digested with the methylation insensitive restriction enzyme MspI (NEB). Ends of each restriction fragment were filled in and a 3′ adenosine was added with Klenow Fragment (3′→5′ exo-minus; NEB). Methylated paired-end Illumina adapters were ligated to the ends of the DNA fragments using T4 DNA Ligase (NEB). Fragments between 105 bp and 185 bp were purified by agarose gel extraction. The purified fragments were treated with sodium bisulfite and then amplified by PCR with long-range PCR conditions and Platinum Taq Polymerase (Invitrogen). The final PCR products were sequenced on Illumina GAIIx machines. All of the sequence data that is presented is of high quality with average quality scores of more than 25 for each cycle. Sequence data for the cell lines as well as the kidney and skeletal muscle tissue as available through the UCSC genome browser's [33] ENCODE DNA methylation track: HAIB Methyl RRBS. Sequence data for the primary blood leukocytes can be found under Gene Expression Omnibus (GEO) submission GSE30253.

### Computational discovery of ASM

All analysis was performed using the February 2009 (GRCh37/hg19) build of the human genome. To determine instances of ASM, we first converted all cytosines in sequence reads to thymidines. We did the same to the reference genome sequence and then aligned the converted sequence reads to the converted reference sequence using Bowtie [36] to look for unique alignments. We required the read be uniquely aligned to only the best position in the converted reference, and that the reference position be the best and unique alignment to itself across the reference genome. We then identified the CpG positions in the unconverted reference sequence, and calculated the fraction of original sequence reads that have a C in that position. We performed further analysis only on CpGs with at least 14× read depth coverage. Bowtie also identifies mismatches between the reads and the reference, so we used it to look for common mismatches in the alignments to identify SNPs. We required at least 7 instances of the same mismatch and also required that the mismatch be found in at least 10% of the total reads that cover that position. We did not use an existing software tool to identify SNPs because the bisulfite reads do not align to a standard genome and all of the reads that cover a potential SNP start at one particular position due to the restriction digest. When analyzing the distribution of SNPs, we found that ASM SNPs that involve a reference G are the most prevalent, which is due to CpG-disrupting SNPs (Table S5). The next most prevalent SNPs involve a reference T, which is most likely due to extra Ts in the reference genome, since both Cs and Ts are represented as Ts in our bisulfite reference genome.

For each called SNP, we determined whether the SNP was heterozygous by requiring that at least 7 sequence reads contain the reference allele. Then for each SNP-CpG pair that is present in the same 36 bp read, we calculated the amount of methylation on the variant allele and the reference allele. To test association of the SNP and the methylation status of the CpG, we performed a Fisher's Exact Test and calculated q-values [37] to assess false discovery rates for each SNP-CpG pair. To look at whether ASM SNPs were from dubious SNPs at the lower quality ends of sequence reads we determined the median distance between SNPs and CpGs for SNP-CpG pairs that exhibit ASM and those that do not. We found that the median distance was 8 bp in each class.

### ASE and ASM validation

For Sanger sequencing validation, bisulfite PCR, cloning and Sanger sequencing was performed as previously described [38]. For allele-specific methylation validation a Fisher's exact test was used to determine significance with a p-value cutoff of 0.05. For allele-specific expression we compared the proportion of reads coming from each allele in the genomic DNA clones to the proportion of reads coming from each allele in the cDNA clones. A binomial test was used to determine significant allele-specific expression with a p-value cutoff of 0.05.

### CpG island, conservation, and gene feature discovery

CpG island locations [39], phyloP [40] scores (Mammalian Cons – phyloP46wayPlacental) and RefSeq [41] exon locations were downloaded from the UCSC genome browser [42].

### Clustering and linear model of DNA methylation and genotype

Average linkage hierarchical clustering based on Euclidean distances was performed on the top 237 most varying CpGs as well as all CpGs that exhibited ASM across the 8 buffy coat samples using the software package Cluster 3.0 [43]. TreeView [44] was used to visualize the data.

A linear model relating genotype and methylation levels was constructed in R. A CpG-SNP pair was analyzed only if the CpG had a standard deviation in percent methylation of at least 25 across family members and the SNP exhibited at least two different genotypes. Genotype values were assigned as follows: 0 = homozygous reference allele, 0.5 = heterozygous, and 1 = homozygous variant allele. We required at least 25 reads covering each SNP to assure that there were no errors in genotype calls. For each CpG-SNP pair, the percent methylation was regressed on the genotype value. ANOVA was then performed to calculate a p-value for each model. We then calculated q-values to assess false discovery rates and considered any model with a false discovery rate less than 5% to be significant.

### Discovery of ASE

RNA-seq was performed as previously described [45] and the analysis is discussed in greater detail elsewhere (T.E.R., et al. manuscript in preparation). To discover ASE, we first created and aligned RNA-seq reads to RefSeq transcripts assembled from a modified genome sequence that contains both haplotypes of GM12878. We obtained SNP and haplotype information from the 1000 Genomes Project [46]. After alignment, the number of reads aligned to each copy of each transcript was calculated. Then a binomial test was used to determine significance and false discovery rates [47] were calculated in R. Transcripts with a false discovery rate less than 5% were considered to harbor allele-specific expression. We used a cutoff of 25 reads, so that at least an 80% bias towards one allele could be detected as significant.

## Supporting Information

Dataset S1 ASM events in each sample.
(TXT)

Figure S1 Comparison of ASM in clonal cell lines derived from the lymphoblastoid line GM12878 with different inactive X chromosomes. Each plot shows the difference in percent methylation of the reference allele and the variant allele for ASM events from two clonal cell lines. Each black circle represents an autosomal ASM event and each red circle represents an X chromosome ASM event. When comparing a line with a paternal inactive X chromosome (Xi-pat) vs. a line with maternal inactive X chromosome (Xi-mat) an average of 59.7% of X chromosome ASM events switch which allele is more often methylated. The results are consistent with DNA methylation being associated with X chromosome inactivation.
(PDF)

Table S1 Prevalence of ASM in each sample.
(XLS)

Table S2 Allele-specific expression of *ATRX* and *TBL1X*, genes expressed from the active X chromosome, indicate that two clones inactivate the maternally inherited X chromosome and two clones inactivate the paternally inherited X chromosome.
(XLS)

Table S3 SNP coverage by category.
(XLS)

Table S4 Overlap between ASM and ASE.
(XLS)

Table S5 Distribution of nucleotides in ASM SNPs.
(XLS)
