# Supplementary figures and images for: Analysis of DNA Methylation in a Three-Generation Family Reveals Widespread Genetic Influence on Epigenetic Regulation
Source: PLoS Genet. 2011 Aug 11;7(8):e1002228. doi: 10.1371/journal.pgen.1002228 (PMC3154961; doi:10.1371/journal.pgen.1002228)

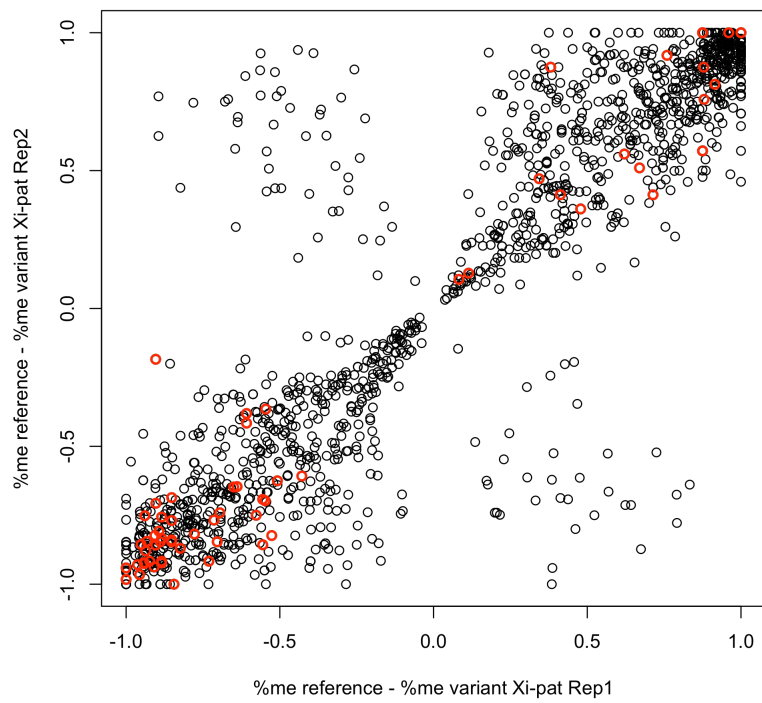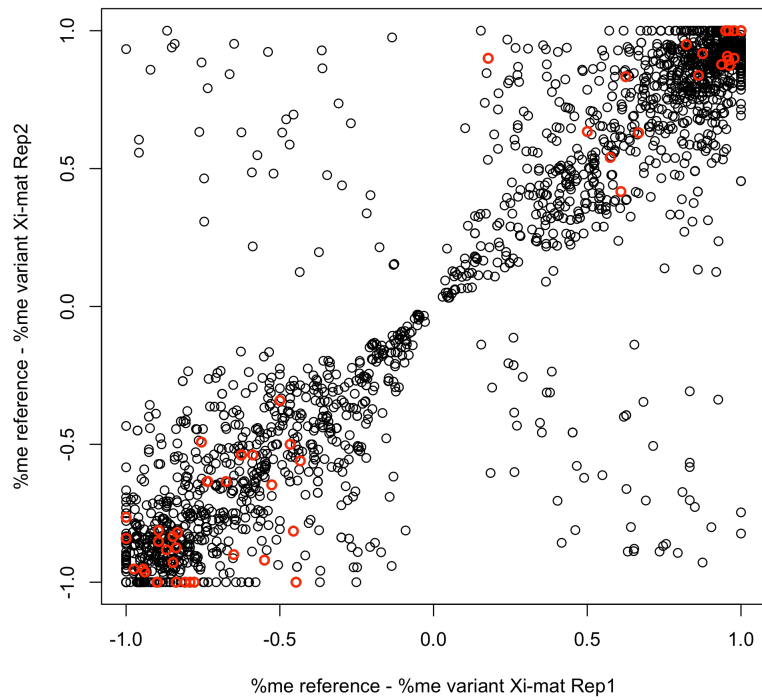

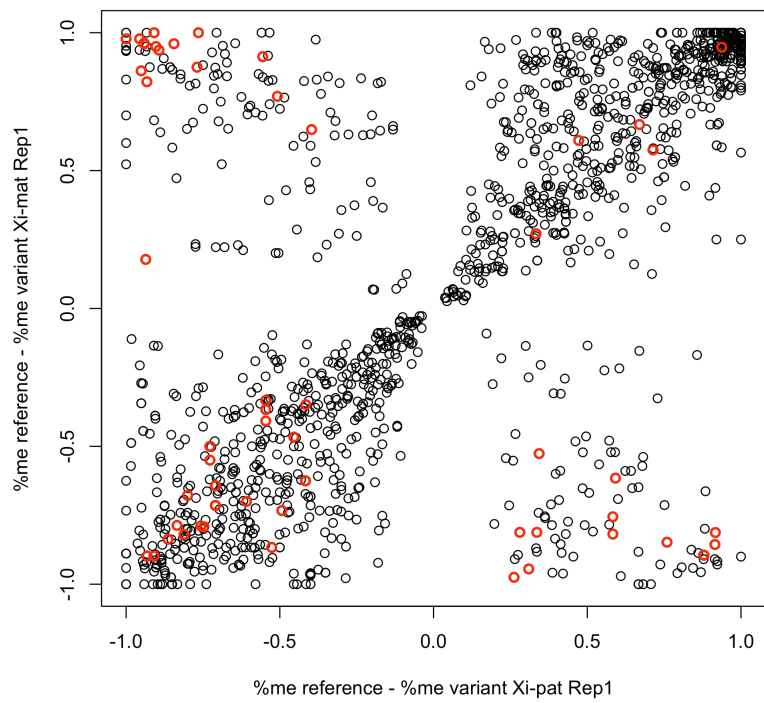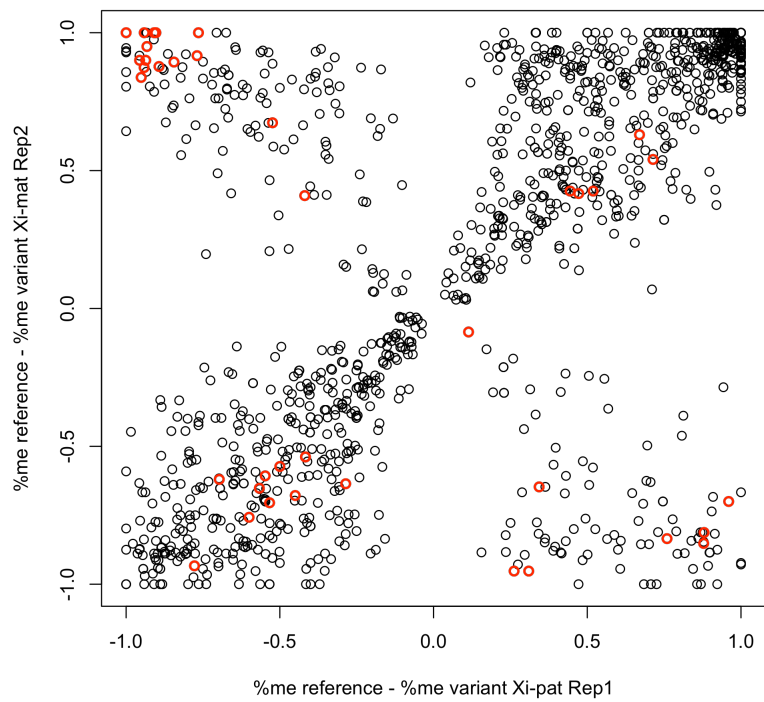

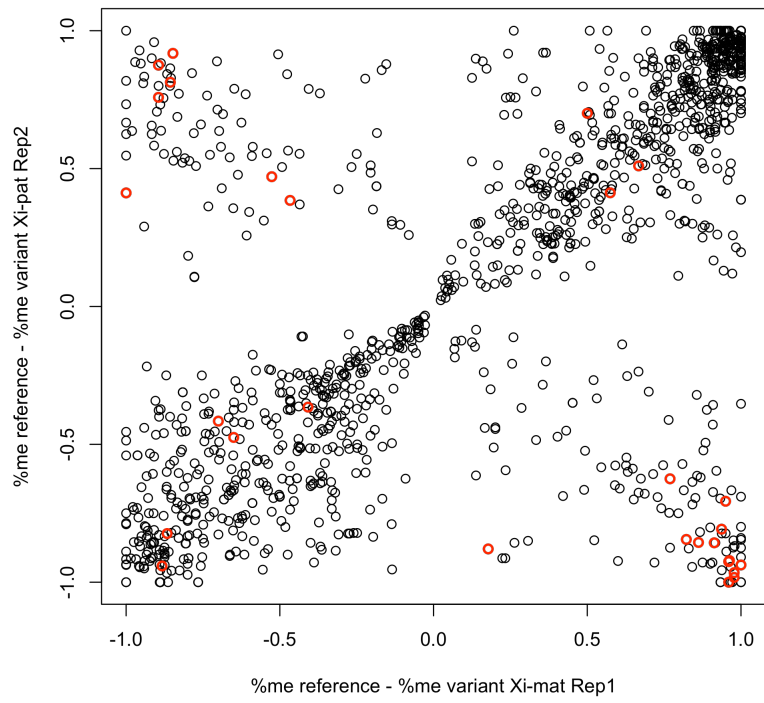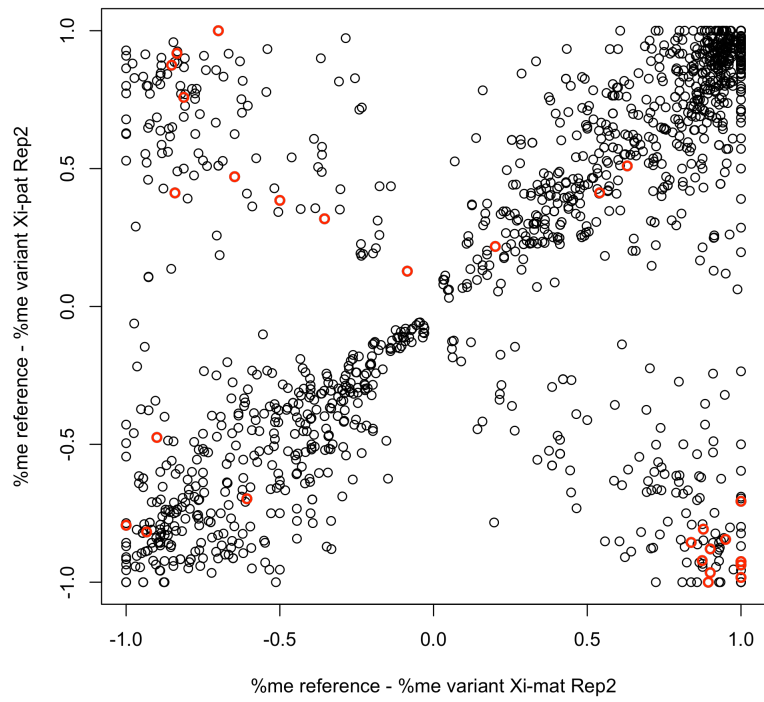

Supplement: Figure S1 — Comparison of ASM in clonal cell lines derived from the lymphoblastoid line GM12878 with different inactive X chromosomes. Each plot shows the difference in percent methylation of the reference allele and the variant allele for ASM events from two clonal cell lines. Each black circle represents an autosomal ASM event and each red circle represents an X chromosome ASM event. When comparing a line with a paternal inactive X chromosome (Xi-pat) vs. a line with maternal inactive X chromosome (Xi-mat) an average of 59.7% of X chromosome ASM events switch which allele is more often methylated. The results are consistent with DNA methylation being associated with X chromosome inactivation. (PDF) [file pgen.1002228.s002.pdf]
